# *Toxoplasma gondii* Infection in the Brain Inhibits Neuronal Degeneration and Learning and Memory Impairments in a Murine Model of Alzheimer's Disease

**DOI:** 10.1371/journal.pone.0033312

**Published:** 2012-03-21

**Authors:** Bong-Kwang Jung, Kyoung-Ho Pyo, Ki Young Shin, Young Sang Hwang, Hyoungsub Lim, Sung Joong Lee, Jung-Ho Moon, Sang Hyung Lee, Yoo-Hun Suh, Jong-Yil Chai, Eun-Hee Shin

**Affiliations:** 1 Department of Parasitology and Tropical Medicine, Institute of Endemic Diseases, Seoul National University College of Medicine, Seoul, Republic of Korea; 2 Department of Pharmacology, Seoul National University College of Medicine, Seoul, Republic of Korea; 3 Department of Neuroscience, School of Dentistry, Seoul National University, Seoul Republic of Korea; 4 Seoul National University Boramae Medical Center, Seoul, Republic of Korea; 5 Seoul National University Bundang Hospital, Seongnam, Republic of Korea; Charité, Campus Benjamin Franklin, Germany

## Abstract

Immunosuppression is a characteristic feature of *Toxoplasma gondii*-infected murine hosts. The present study aimed to determine the effect of the immunosuppression induced by *T. gondii* infection on the pathogenesis and progression of Alzheimer's disease (AD) in Tg2576 AD mice. Mice were infected with a cyst-forming strain (ME49) of *T. gondii*, and levels of inflammatory mediators (IFN-γ and nitric oxide), anti-inflammatory cytokines (IL-10 and TGF-β), neuronal damage, and β-amyloid plaque deposition were examined in brain tissues and/or in BV-2 microglial cells. In addition, behavioral tests, including the water maze and Y-maze tests, were performed on *T. gondii*-infected and uninfected Tg2576 mice. Results revealed that whereas the level of IFN-γ was unchanged, the levels of anti-inflammatory cytokines were significantly higher in *T. gondii*-infected mice than in uninfected mice, and in BV-2 cells treated with *T. gondii* lysate antigen. Furthermore, nitrite production from primary cultured brain microglial cells and BV-2 cells was reduced by the addition of *T. gondii* lysate antigen (TLA), and β-amyloid plaque deposition in the cortex and hippocampus of Tg2576 mouse brains was remarkably lower in *T. gondii*-infected AD mice than in uninfected controls. In addition, water maze and Y-maze test results revealed retarded cognitive capacities in uninfected mice as compared with infected mice. These findings demonstrate the favorable effects of the immunosuppression induced by *T. gondii* infection on the pathogenesis and progression of AD in Tg2576 mice.

## Introduction

*Toxoplasma gondii* (*T. gondii*) is a protozoan parasite that commonly infects humans and animals [1]. Humans are generally infected by ingesting oocysts released in cat feces or by consuming undercooked meat containing tissue cysts. Following ingestion, bradyzoites and sporozoites released from cysts and oocysts invade intestinal cells and convert to tachyzoites [1], which when disseminated via blood or the lymphatic system to remote organs induce acute or chronic inflammatory responses. Furthermore, during the chronic stage, the brain is the most commonly affected site [2]. *T. gondii* is a serious pathogen that can invade vital organs, but usually the infection is mild and asymptomatic in immunocompetent hosts. Possible clinical manifestations include lymphadenopathy, myocarditis, hepatitis, sepsis syndrome, retinochoroiditis, and encephalitis [1]. However, normally the infection becomes chronic, remains latent in the brain, and elicits life-long immunity against toxoplasmosis [1].

Immune responses to *T. gondii* infection differ during the proliferative (acute phase) and dormant (chronic and latent phase) stages and depend on the virulence of the parasite strain, for example, RH is a highly virulent strain (type I) whereas ME49 is avirulent (type II). The acute phase is characterized by marked elevations in serum Th1 cytokine levels, such as, of IFN-γ, TNF-α, IL-12, and IL-18, and is followed by a lethal outcome in mice. On the other hand, non-lethal infection is characterized by modest elevations in Th1 cytokine levels that led to the control of *T. gondii* infection and minimal damage to the host [3]. In particular, ME49 is an avirulent strain that can exist in the brain for a considerable time by suppressing immune responses in the central nervous system (CNS) [3], [4]. During the course of *T. gondii* infection, the levels of anti-inflammatory cytokines, like IL-10 and TGF-β, increase in brain tissues, whereas the productions of inflammatory mediators, such as, nitric oxide (NO), decrease [4], [5], [6]. Furthermore, these anti-inflammatory responses may prevent tissue injury and establish a chronic state of host-parasite equilibrium [4], [5], [6].

Notably, the neuronal degeneration induced by neuroinflammation is not a common finding in the brains of mice chronically infected with *T. gondii* [7]. Similarly, chronic or latent infections by infectious agents in the CNS may delay the onset of neurodegenerative changes related to nerve inflammation [8]. Regarding neuroinflammation in *T. gondii* infection, NO production is known to be an important regulator and indicator. For example, NO production was found to be significantly down-regulation when conditioned medium of a *T. gondii*-infected astrocyte culture was added to a microglia culture [7], which suggests that the immune responses triggered by *T. gondii* infection can reduce inflammatory response in the host brain and prevent neuronal degeneration.

The neuronal degeneration induced by neuroinflammation is known to play a key role in the pathogenesis of chronic neurodegenerative diseases [9], [10], and in particular, Alzheimer's disease (AD) is the most common cause of dementia in the elderly causing progressive and permanent reductions in learning, memory, and cognitive abilities [10]. The pathogenesis of AD is characterized by widespread neuronal degeneration, involving synaptic and neuronal loss, and the formations of extracellular neuritic plaques containing β-amyloid peptides and intracellular neurofibrillary tangles [10]. Histologically, AD brain tissues show increased numbers of reactive microglia [11], which when exposed to inflammatory stimuli express the inducible form of NO synthase (iNOS), and thus, increase NO production [12]. Moreover, adjacent neuronal cells are extremely susceptible to the toxic effects of NO, and this sensitivity plays a central role in the pathogenesis of neurodegenerative diseases [12], [13].

Microglial cells also augment inflammatory responses by releasing various mediators, such as, cytokines, reactive oxygen species, complement factors, neurotoxic secretary products, and free radicals [7], [8], [9], [10], [11], [12], [13], [14], [15], and many of these mediators are known to stimulate amyloid precursor protein (APP) deposition and contribute to neuronal death in AD [7], [8], [9], [10], [11], [12], [13], [14], [15]. Eventually, microglial cell activation can establish a vicious cycle of inflammatory mediator release and the stimulation of APP production [15]. In this respect, we considered that it would be interesting to probe the relationship between host immune response against the long-lived and latent pathogen *T. gondii* in the brain and the progression of age-related neurodegenerative disorder.

Investigations on the possible link between *T. gondii* infection and degenerative central nervous system (CNS) disease in man have been mainly limited in the serology testing of *T. gondii*-specific antibody [16], [17], [18]. Recently, such studies have been conducted in AD patients, PD patients, and patients with other psychiatric conditions. For example, in one study, the seropositivity rate for *T. gondii*-specific IgG was found to be more in patients with AD than in healthy controls (44.1% versus 24.3%) [16]. In another study, 42 patients with obsessive-compulsive disorder (OCD) and 52 patients with Parkinson's disease (PD) also showed significantly higher titers in seropositivity than controls [17], [18]. As a result, the authors suggested that *T. gondii* infection may be involved in the pathogenetic mechanisms of CNS diseases [16], [18]. However, in those study the anti-*T. gondii* IgG titers shows IgG levels examined at that time, and thus, do not suggest that a causal relationship exists between toxoplasmosis and the etiology of psychiatric illness and dementia [19]. Because the causal relationship between toxoplasmosis and the etiology of CNS diseases cannot be simply determined, experimentation is needed under known conditions, that is, with information on times between infection and disease onset.

In the present study, we undertook to determine whether *T. gondii* infection is involved in the neuroinflammation and neurodegeneration mechanisms of dementia. We hypothesized that latent *T. gondii* infection in the mouse brain may induce immunosuppression to escape host immune attack, and thus, act to suppress the pathogenesis of AD. Thus, the objective of the present study was to investigate the effect of the inhibition of neuroinflammation by *T. gondii* on the progression of AD. For this purpose, we used Tg2576 mice (an accepted model of AD) infected with an avirulent strain (ME49). Degrees of neuronal protection in brains were determined histologically, and immune responses were examined in brain tissues and BV-2 microglial cells. In addition, the water and Y-maze behavioral tests were used to quantify learning and memory abilities and cognitive functions.

## Results

### *T. gondii* infection in Tg2576 AD mice inhibited neuronal degeneration and induced IL-10 and TGF-β

**Figure 1:**
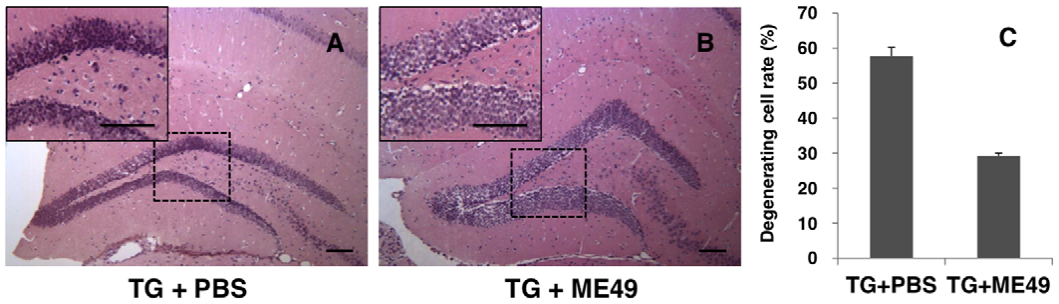
*T. gondii* infection inhibited spontaneous neuronal degeneration in Tg2576 AD mice. Histological changes in the hippocampal formation of Tg2576 AD mice were observed by H&E staining, and changes in *T. gondii*-infected (B; ME49) and uninfected mice were compared (A; PBS). Neuronal death, represented by eosinophilic neurons (PBS, ×100), was remarkably lower in the hippocampal region of infected Tg2576 mice (B; ME49) than in Tg2576 mice (A; PBS), which showed spontaneous neuronal degeneration (×100). Scale bar = 100 µm. Results are represented as percentages of degenerative cells among all cells in dentate gyrus of hippocampal formation (C).

To determine the neuronal damage caused by *T. gondii* infection, histopathologic changes in the hippocampal region were examined by hematoxylin and eosin (H-E) staining in Tg2576 AD mice infected or not infected by *T. gondii*. As shown in Figure 1, neuronal death (eosinophilic neurons) was remarkable in the hippocampal region of phosphate-buffered saline (PBS)-treated uninfected mice (Figure 1A, progressive AD mice as a control), whereas infected Tg2576 mice (Figure 1B) and infected wild type mice (data not shown) exhibited few eosinophilic neurons in the same region. Progressive AD mice (TG+PBS) showed numerous dead neurons ((57.7±2.5)%) in the dentate gyrus at 9 months after birth (Figure 1C), whereas *T. gondii*-infected Tg2576 mice (TG+ME49) showed relatively few dead neurons ((29.2±0.81)%) (Figure 1C)

**Figure 2:**
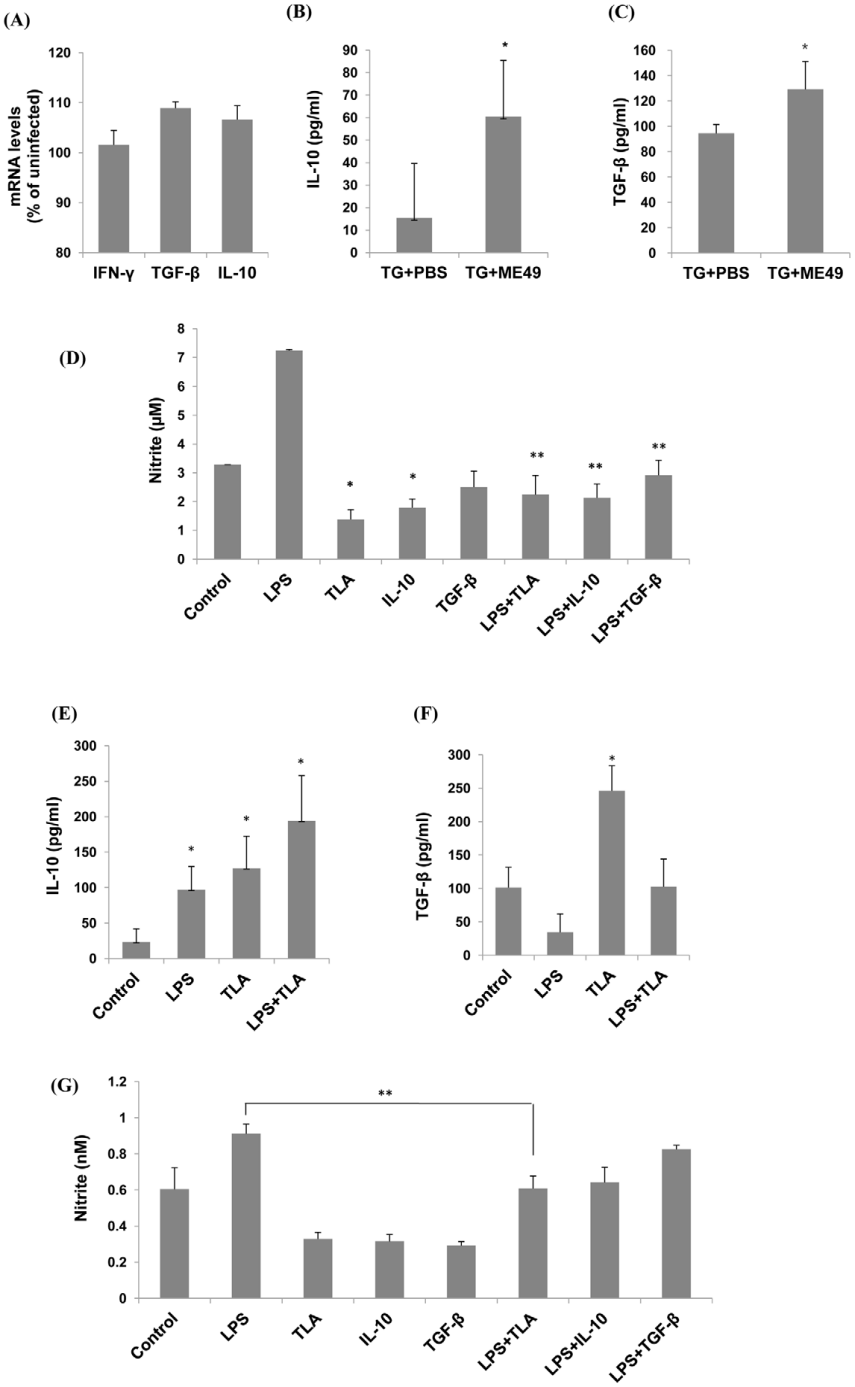
*T. gondii* induced an inflammation-suppressing immune environment *in vivo* and *in vitro*. (A) The mRNA expressions of inflammation-suppressing cytokines (IL-10 and TGF-β) were higher in *T. gondii* (Me49)-infected Tg2576 mice than in their uninfected littermates (A). mRNA levels are presented as percentages of cytokine levels in infected mice versus uninfected mice. Furthermore, IL-10 and TGF-β cytokines were significantly higher in the brain tissues of infected mice than in those of uninfected mice (P<0.05) (B & C). Nitrite levels suggested the production of nitric oxide (NO), an inflammatory mediator related to neuronal death. Primary cultured microglial cells were prepared from wild type mice (C57BL/6 mice) and tested for nitrite production (D). Microglial cells cultured from brain tissues showed a significant decrease in nitrite production when TLA or anti-inflammatory cytokines were added (D). To investigate the possibility that these cytokines were produced by microglial cells, BV-2 cells were cultivated with LPS and/or *T. gondii* lysate antigen (TLA) (E & F). BV-2 microglia treated with TLA significantly increased the productions of IL-10 (E) and TGF-β (F) in the presence or absence of LPS. BV-2 cells were incubated for 12 h in the presence of TLA, LPS, IL-10, or TGF-β, and the supernatants obtained were analyzed for nitrite (G). The results obtained showed that nitrite concentrations were lower in TLA-, IL-10-, or TGF-β-treated cells than in non-treated control cells. In particular, the nitrite concentration increase induced by LPS was significantly lowered by co-treatment with TLA (P<0.05).

Because neurodegeneration is related to immune balance between inflammatory mediators and inflammation-suppressing cytokines, the mRNA levels of IFN-γ, IL-10, and TGF-β were examined in *T. gondii*-infected Tg2576 mice by quantitative real-time PCR (Figure 2A). IFN-γ mRNA levels were found to be no different in infected and uninfected Tg2576 mice, but IL-10 and TGF-β mRNA levels were higher in infected mice than in uninfected control mice (Fure 2A).

Because the anti-inflammatory cytokines, IL-10 and TGF-β, are produced by microglial cells for neuroprotection after traumatic injury or stroke [20], we examined the secretion of these cytokines using *T. gondii*-infected Tg2576 mouse brain tissues (Figure 2B, C) and BV2 cells (a microglial cell line; Figure 2E, F). IL-10 and TGF-β cytokine levels were found to be significantly higher in infected Tg2576 mouse brain tissues (TG+ME49) than in uninfected control mouse tissues (TG+PBS) (p<0.05) (Figure 2B–C). Furthermore, levels of IL-10 and TGF-β in BV2 cells cultured *in vitro* were found to be increased remarkably by the addition of *T. gondii* lysate antigen (TLA), which concurred with our findings in *T. gondii*-infected mouse brains (Figure 2B–C).

When exposed to inflammatory stimuli, microglial cells produce the inducible form of NO synthase (iNOS), and the NO subsequently produced is critically required to construct protective host responses against foreign pathogens. However, this immune response eventually damages host tissues, and in particular, NO is a key mediator of glia-induced neuronal death [12]. In the present study, primary cultured microglial cells were prepared from wild type mice (C57BL/6), and incubated with TLA, recombinant IL-10, or recombinant TGF-β (Figure 2D). As was expected, 100 ng/ml LPS increased nitrite production by cells, whereas the addition of 50 µg/ml TLA, recombinant IL-10, or recombinant TGF-β decreased nitrite production. Furthermore, the effect of TLA on nitrite production was also observed in the presence of LPS (Figure 2D). Microglial cells from primary cultured brain tissues showed significantly less nitrite production when TLA was added, and the addition of TLA decreased nitrite production under conditions of LPS stimulation (Figure 2D).

Similarly, anti-inflammatory cytokines and NO levels were measured after treating BV2 cells with TLA (Figure 2E, F, G). The addition of LPS increased nitrite production by cells, whereas the addition of TLA, IL-10, or TGF-β decreased nitrite levels (Figure 2G). Observed decreases in nitrite production were similar when cells were treated with TLA, IL-10, or TGF-β. However, when they were co-treated with LPS, the reduced nitrite level elicited by TLA was significantly greater than the levels elicited by IL-10 and TGF-β (Figure 2G; p<0.05). The above results show that the productions of anti-inflammatory cytokines (IL-10 and TGF-β) and reduced nitrite production were observed under all experimental conditions (*T. gondii*-infected Tg2576 mice and TLA stimulated primary cultured microglia and BV-2 cells).

### *T. gondii* infection inhibited β-amyloid plaque formation in the cortex and hippocampus of Tg2576 mice

**Figure 3:**
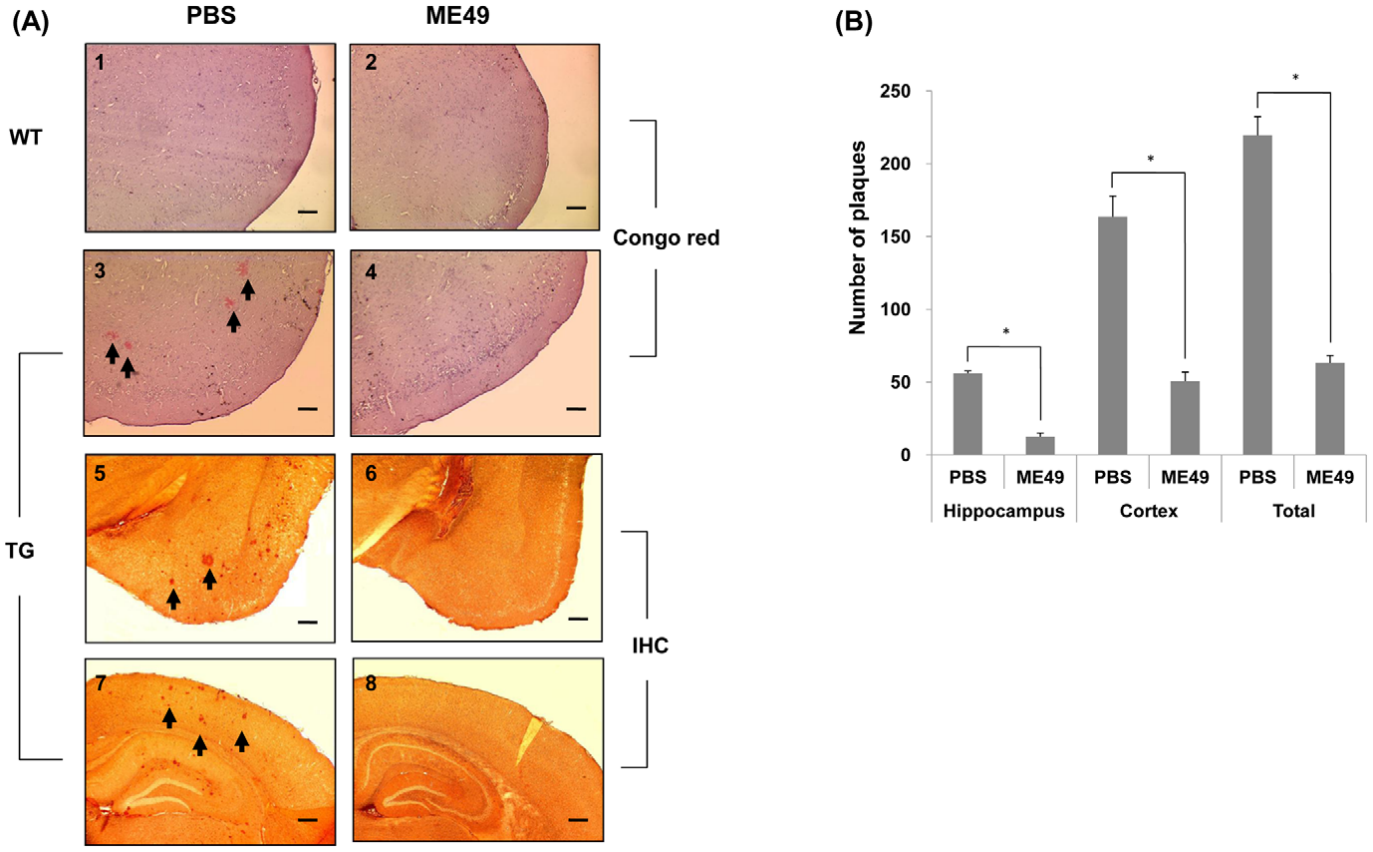
Deposition of beta-amyloid plaque in the brains of mice with or without *T. gondii* infection. Differences between β-amyloid deposit levels in *T. gondii*-infected and non-infected mice (PBS-treated mice) were examined in the cortex and hippocampus regions using Congo red and by immunohistochemical staining with 6E10 antibody. Congo red staining was performed on; uninfected (PBS-treated) wild type (A1), *T. gondii*-infected wild type (A2), uninfected Tg2576 (A3), and infected Tg2576 (A4) mouse groups. Wild type mice with or without *T. gondii* infection showed no amyloid plaque (A1, A2) at 9 mo after birth, whereas PBS-treated Tg2576 mice showed many Congo red-stained regions in the cortex (A3). However, no amyloid plaque was observed in *T. gondii* infected Tg2576 mouse brain tissues (A4). Immunostaining results of cortex and hippocampus concurred with Congo red findings (A5, A6, A7, A8). (×40, Scale bar = 200 µm).). In addition, numbers of plaques in cortex and hippocampus were counted using a color digital camera attached to a microscope and Image J software, and the results obtained showed that numbers of plaques were significantly less in the hippocampus and cortex of *T. gondii*-infected Tg2576 mice (B).

Previous reports have indicated that inflammatory mediators stimulate amyloidogenesis in AD by inducing APP production and β-secretase expression [15], [21]. In the present study, we investigated whether suppression of neurodegeneration and the anti-inflammatory cytokine increase observed in *T. gondii*-infected Tg2576 mice affect amyloidogenesis. The relationship between infection and β-amyloid deposition was examined by Congo red staining and immunohistochemistry in the brains of Tg2576 mice using β-amyloid specific 6E10 antibody, which specifically recognizes the 1–17 amino acid sequence of β-amyloid peptides (Figure 3). Furthermore, whereas the brain tissues of wild type mice did not contain β-amyloid plaques by Congo red staining (Figure 3A1–2), Tg2576 mice typically generated considerable amounts of amyloid plaque (Figure 3A3, 3B), but *T. gondii*-infected Tg2576 mice exhibited obviously lower amounts of plaque (Figure 3A4, 3B). We also evaluated β-amyloid protein deposition by immunohistochemical (IHC) staining using 6E10 antibody (Figure 3A5, 6, 7, 8). It was found extracellular β-amyloid plaques in the cortex (Figure 3A5) and hippocampal region (Figure 3A7) of uninfected Tg2576 mice were remarkably greater than in *T. gondii*-infected Tg2576 mice (Figure 3A6, 3A8), and that the number of plaques was significantly lower in *T. gondii*-infected Tg2576 mice in both hippocampus and cortex (Figure 3B).

### *T. gondii* infection attenuated learning and memory impairments in Tg2576 mice

*T. gondii* infection was found to induce the productions of IL-10 and TGF-β in brain and to suppress nitrite production in response to TLA, indicating the local inductions of anti-inflammatory responses in brain. In addition, β-amyloid plaque depositions were found to be reduced in the cortex and hippocampus of infected mice. These findings raised the question as to whether progressive impairments of learning and memory in Tg2576 mice could be reduced by the anti-inflammatory responses induced by *T. gondii* infection.

**Figure 4:**
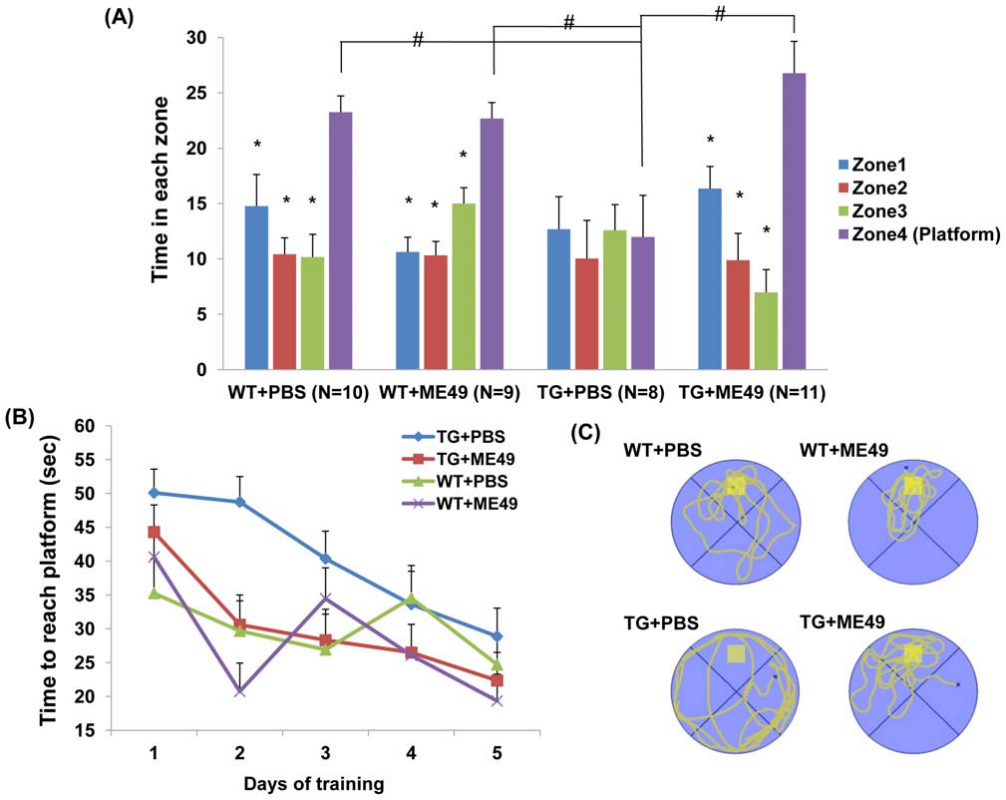
Evaluation of learning and memory using the Morris water maze test. Differences in learning and memory between wild type (WT) and Tg2576 mice (TG) at 9 mo of age were examined using the Morris water maze test. (A) In a 60 s probe trial, the ability of uninfected (PBS-treated)-Tg2576 mice (TG+PBS) to find the training quadrant (zone 4, which contained the platform) was significantly less than those of mice in the other experimental groups (A; p<0.0001). On the other hand, *T. gondii*-infected Tg2576 mice (TG+ME49) performed as well as wild type mice (B). Representative swimming paths of mice during the probe trial (platform removed) were as follows (C); TG+PBS mice seemed unaware of the platform position, whereas TG+ME49 mice and wild-type mice (WT+PBS and WT+ME49) remained in the vicinity of the platform. The yellow box in the figure indicates the hidden platform.

Based on these findings, we used the Morris water maze and the Y-maze behavioral tests to quantify learning and memory functions, as previously described [22]. In the water maze test, Tg2576 mice infected with *T. gondii* were found to differ significantly from uninfected (PBS-treated) mice (p = 0.0055, F = 4.49; Figure 4A). However, no difference was found between *T. gondii*-infected and uninfected wild type mice (Figure 4A). In the probe test at 48 hours after final spatial training, *T. gondii*-infected Tg2576 mice stayed significantly longer in zone 4 than in other zones (zones 1–3) (Figure 4A). Mean durations in each zone for *T. gondii*-infected Tg2576 mice in the water maze test were as follows; zone 4 (platform), 26.77 s; zone 1, 16.36 s; zone 2, 9.87 s; and zone 3, 6.98 s (Figure 4A). However, uninfected Tg2576 mice exhibited a different pattern, suggesting loss of memory (zone 1, 12.69 s; zone 2, 10.04 s; zone 3, 12.58 s; and zone 4, 11.97 s). This result was confirmed by measuring the mean times required to reach the platform (a measure of learning and memory) (Figure 4B), and by investigating swimming paths in zone 4 during the last probe trial (platform removed) (Figure 4C).

**Figure 5:**
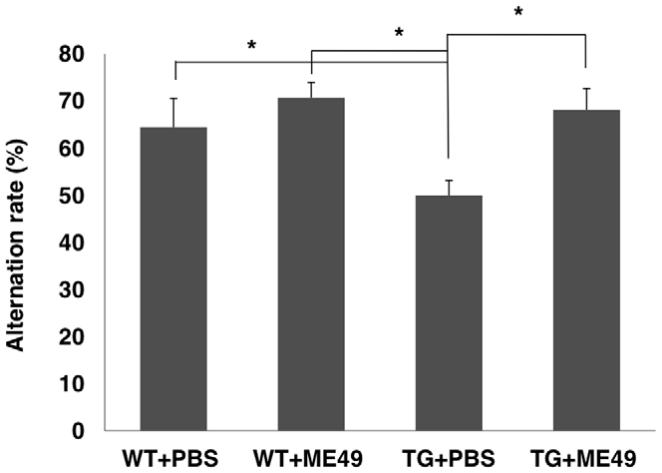
Evaluation of learning and memory using the Y-maze test. Tg2576 and wild type mice with or without *T. gondii* infection were tested. A significant difference in success rates was observed between uninfected Tg2576 mice (Tg+PBS) and the other experimental groups (WT+PBS, WT+ME49, Tg+ME49) (P<0.01).

The Y-maze test was also conducted to evaluate learning and memory functions, as described previously [23] (Figure 5). The success rate of *T. gondii*-infected mice in the Y-maze test was significantly greater than for uninfected mice (p<0.01, by one-way ANOVA and Tukey's post hoc test). Accordingly, Morris water maze and Y-maze testing showed that *T. gondii* infection inhibited spontaneous memory functional impairments in Tg2576 mice (Figure 5).

The passive avoidance test was performed to confirm the effect of *T. gondii* infection on wild type and Tg2576 mice (Figure S1). Times taken to enter the dark chamber (latencies) were measured for 300 sec (retention trial). *T. gondii*-infected Tg2576 mice were found to have greater latencies (Figure S1), which means the *T. gondii* infection in AD model mice (Tg2576) inhibited memory deterioration, as was observed for the Morris water maze and Y-maze behavioral tests.

## Discussion

*T. gondii* is a zoonotic protozoan that can infect many vertebrates. It has a worldwide distribution, and is the causative agent of human and animal toxoplasmosis [2]. According to a survey conducted in the USA in 1988–1994, *T. gondii* seroprevalence in the overall age-adjusted population was 22.5%, and it showed a gradual increase with age [24]. Furthermore, France and several other European countries, Latin America, and sub-Saharan Africa have even higher seroprevalences than the USA [25].

*T. gondii* has two developmental stages in man, that is, tachyzoites (trophozoites during acute infection) and bradyzoites (cysts during chronic infection), and these have different pathogenic consequences [2]. Tachyzoites disseminated via blood or the lymphatic system to different organs during acute stage disease cause toxoplasmosis, which is characterized by lymphadenopathy and reticular cell hyperplasia [2]. However, during the chronic, latent stage, the brain is the most commonly affected [2]. Furthermore, during this stage, the infection is likely to be accompanied by nerve degeneration, which progresses with age. In this respect, *T. gondii* is an important infectious agent during the course of neuronal degenerative diseases, such as, AD. However, the relationships between chronic, latent *T. gondii* infection and age-related neuronal degenerative diseases have not been determined. Since most cases of primary *T. gondii* infection are asymptomatic and the parasite has established immune privilege in chronically infected host tissues, such as, in the brain [2], the relationships between neurodegenerative diseases, such as, AD, and *T. gondii* infection have been overlooked.

*T. gondii* is classified into three clonal lineages by virulence [3]. The type I genotype is highly virulent to mice, whereas the lethalities of the type II and III strains are substantially lower. Type II strains, which include ME49, are highly prevalent in animals, and are also associated with toxoplasmosis in man [3]. The lethality of *T. gondii* in mice is largely determined by strain genotype. Lethal infections are mediated by a strong Th1 cytokine response, such as, by IFN-γ, IL-12, TNF-α, and IL-18, whereas non-lethal type II ME49 infections are controlled by the modest inductions of Th1 cytokines [3]. The overstimulation of host immune responses that are normally required for protection is an important immunological feature in acute toxoplasmosis [3]. In contrast, during latent infections, *T. gondii* contributes to the control of host immune response in a manner that results in immunosuppression [5], [7], [26]. In fact, several *in vitro* studies have shown that *T. gondii* triggered immune responses reduce inflammatory responses and prevent neuronal degeneration [5], [7]. Thus, the reduction of neuroinflammation by immune modulation could alter the disease onset and progression of AD [9], [10], [12], [13], [14], [15]. However, although the authors of these reports suggested that *T. gondii* infection in the brain reduces neuroinflammation, the effects of *T. gondii* infection in the development of neurodegenerative disorders, such as, AD, have not been examined.

In recent studies, it has been found that more than 40% of patients with a severe CNS disease have anti-*T. gondii* IgG [16], [17], [18]. In a Turkish study of such patients, *T. gondii* infection was screened for based on seropositivity against *T. gondii* immunoglobulin regardless of symptom onset. Accordingly, results would be interpreted to mean that psychiatric and dementia patients have life styles that differ from those of healthy controls [19], for example, patients with impaired social and occupational functions are more likely to stay at home, and if they owned cats, would be at greater risk of being exposed to *T. gondii* [19]. Thus the causal relationship between toxoplasmosis and the etiology of certain CNS diseases, such as, dementia, cannot be easily determined [19]. To overcome these types of problems associated with human research, animal research is required because it can provide information regarding temporal relations between infection and disease onset.

In the present study, we focused on the effects of chronic, latent *Toxoplasma* infection in the brains of Tg 2576 mice on neurodegenerative changes and inflammation induced genetically. Histopathologic studies showed that the hippocampal region of non-infected Tg 2576 mice exhibited many eosinophilic β-amyloid peptide plaques, and showed that these plaques were less present in the brains of *T. gondii*-infected Tg2576 mice or wild type mice. These results convince us that the neuroprotection observed was due to *T. gondii* infection. Furthermore, our results also suggest that the neuroprotective effects of *T. gondii* extend to learning and memory impairments in Tg2576 mice and possibly to the progression of AD.

In the CNS, microglial cells, astrocytes, and neurons are susceptible to *T. gondii* infection, and in chronic infections, latent cysts are produced in the CNS [27]. Although microglial cells are largely responsible for preventing *T. gondii* proliferation in the brain, they also sometimes produce the anti-inflammatory cytokine IL-10, and facilitate parasite persistence by suppressing immune responses in the CNS [28], [29]. IL-10 is produced mainly by brain mononuclear cells, and inhibits the productions of IL-12, IL-6, IFN-γ, and TNF-α, suggesting that IL-10 has an ameliorative effect on the severe inflammation caused by toxoplasmic encephalitis [30].

Notably, an intraperitoneal injection of lipopolysaccharide (LPS), an inflammation inducing endotoxin, was found to result in memory impairments in mice [31]. In addition, repeated injections of LPS enhanced β-amyloid generation, and conversely, treatment with anti-inflammatory agents suppressed LPS-induced amyloidogenesis, memory dysfunction, and neuronal cell death [31]. In this study, it was also found that inflammation resulted in an accumulation of β-amyloid 1–42 via increased β- and γ-secretase activities accompanied by elevated APP expression and astrocyte activation [31].

In the AD brain, various neuroinflammatory mediators, including complement activators and inhibitors, chemokines, cytokines, radical oxygen species, and inflammatory enzyme systems, are released by microglia, astrocytes, and neurons [15]. Accordingly, anti-inflammatory cytokines, such as, IL-10 and TGF-β, are required to prevent immunopathological effects by inhibiting the chemotactic migrations of these microglial cells toward β-amyloid aggregates and by directly inducing anti-inflammatory responses [15], [32]. Convincing evidence shows that Th2 cytokines have beneficial immunomodulatory effects in toxoplasmosis [28], [29], [30]. Actually, IL-10 is known to play vital roles in the control of inflammatory responses during acute toxoplasmosis, and to inhibit tissue damage during the chronic phase [28], [29], [30]. Furthermore, the high susceptibility of IL-10-knockout mice to *T. gondii* infection has been shown to be mediated by an exacerbated inflammatory process not caused by parasite proliferation [33]. The inflammation process is an important countermeasure to infection, but it also damages host tissues. In the long term, harmful inflammations may be eliminated by pathogen's strategy to inhibit host cellular immunity [28], [29], [30]. *T. gondii* provides a good example of this effect.

In the present study, *T. gondii* was found to induce an anti-inflammatory effect by up-regulating IL-10 and TGF-β. Furthermore, *in vitro* treatment of primary microglial cells and BV-2 cells with TLA reduced NO production, which is known to play important roles during neuroinflammation and the pathogenesis of AD. Furthermore, inflammation in the brain may be an important component of the mechanism of dementia and cognitive decline in the elderly. Accordingly, it has been suggested that the inhibition of inflammatory cascades may attenuate amyloidogenic processes, such as, those of AD [34]. Furthermore, because the suppression of NO synthesis was found to prevent cell death and restore lymphocyte proliferation in *T. gondii* (ME49)-infected mice [4], our results support the notion that the inhibition of neuroinflammation by *T. gondii* attenuates the progression of amyloidogenesis in Tg2576 mice.

TGF-β1 and IL-10 are important cytokines that can induce tolerance and suppress exaggerated immune response. In particular, in one study, elevated levels of TGF-ß1 were found to be related to amyloid plaque reductions in the parenchyma, hippocampus, and neocortex of the hAPP Tg mouse brain, and to reduced levels of dystrophic neuritis in aged hAPP Tg mice [35]. Similarly, a decrease in TGF-β signaling in cultured neuroblastoma cells was found to cause neuronal degeneration and to increase levels of secreted Aβ and β-secretase-cleaved soluble APP [36].

Our results also show that *T. gondii*-infected Tg2576 mice exhibited higher levels of the anti-inflammatory cytokines, IL-10 and TGF-β, in brain tissues, and less neuronal death, amyloid plaque deposition, and neurodegeneration than non-infected mice. In one previous study, it was found that *T. gondii*-triggered immune regulations, which included prostaglandin E2 secretion by astrocytes and cAMP-dependent IL-10 secretion by microglia, reduced NO production, and as a result, the authors suggested that *T. gondii* reduced tissue inflammation in the host brain [7]. An *in vivo* study also demonstrated a remarkable increase of IL-10 expression in the brains of *T. gondii*-infected mice [37]. In the present study, *T. gondii* antigen decreased NO production by primary cultured microglia and BV-2 cells, which also suggests that *T. gondii* prevented neuron degeneration in Tg2576 AD model mice.

One interesting observation made in the present study was that *T. gondii* infection attenuated impairments in learning and memory functions in Tg2576 mice, as demonstrated by the water- and Y-maze tests. Furthermore, these results well matched with the observed reduction in NO production and the expressional upregulations of anti-inflammatory cytokines. It is known that Tg2576 mice exhibit an increase in ß-amyloid deposition and memory loss at the time of the appearance of detergent-insoluble ß-amyloid aggregates (ß-amyloid_insol_) 6 months after birth [22]. In the present study, *T. gondii*-infected Tg2576 mice were found to retain the memory capacities of non-AD wild type mice (control mice) according to water- and Y-maze test results. In this respect, our findings agree with those of other investigators, who found that C57BL/6 mice infected with the avirulent ME49 strain showed normal cognitive behaviors despite widespread brain pathologies and sensorimotor deficits [38].

The present study demonstrates effects of chronic, latent *T. gondii* infection on progressive neurodegenerative disease in an AD transgenic mouse model, whereas previous studies has been performed at the early infection stage (within 2 months of infection) [3], [4], [28], [29], [37]. The age of the mice used in the present study and the infection period seem to be more relevant, because *T. gondii* has a long latency period in the host brain [1]. Thus, our results describe the long-lasting (at least 6 months after infection) effects of *T. gondii* infection on mice. In addition, our study highlights the possibility that the neuroprotection induced by *T. gondii* infection before the onset of AD could inhibit the β-amyloid accumulation and neurodegeneration that probably diminish cognitive abilities. Furthermore, we suggest that our results, which obtained using a mouse AD model, are helpful in terms of determining the relationship between *T. gondii* infection and human AD, because most cases of human toxoplasmosis are caused by genotype II, to which ME49 used in the present study belongs [39], [40].

## Materials and Methods

### Experimental Animals

Tg2576 mice (an established animal model of AD) expressing the mutated human APP695 gene [41] were obtained from Taconic (German Town, NY, USA) and were bred by mating male mice with (C57BL6/SJL) F1 females as recommended by suppliers and as described by others [42], [43]. The genotyping was performed by confirming the presence of the human APP695 transgene by PCR analysis of genomic DNA in the mouse tail tips. A pair of primers for detecting human APP695 and PCR condition was as follows. Forward (5′- CTGACCACTCGACCAGGTTCTGGGT-3′) and reverse (5′- GTGGATAACCCCTCCCCCAGCCTAGACCA -3′) primers amplified a 466-bp DNA fragment. The reaction conditions used were; initial denaturation at 94°C for 10 min, 35 amplification cycles [denaturation at 94°C for 1 min, annealing at 55°C for 30 s, and elongation at 72°C for 1 min], followed by one cycle at 72°C for 5 min. Tg2576 mice over-expressing human APP695 containing the “Swedish” mutation have memory deficits and show progressive ß-amyloid plaque deposition with age [42]. Because these mice exhibit a rapid increase in ß-amyloid at around 6 months after birth and the formation of amyloid plaque before 6–9 months, they represent a suitable animal model for examining relationships between ß-amyloid and memory [42].

### Ethics Statement

This study was carried out in strict accordance with the Guidelines for Animal Experiments issued by the Ethics Committee at Seoul National University. The study protocol was approved by the Committee on the Ethics of Animal Experiments at Seoul National University (Permit Number: SNU-080625-1). All surgeries were performed under anesthesia, and all efforts were made to minimize animal suffering.

### *T. gondii* infection

*T. gondii*, ME49 strain, was maintained by injecting cysts isolated from the brains of infected C57BL/6 mice (Orient Bio Animal Center, Seongnam, Gyeonggi-do, Korea) intraperitoneally. To infect Tg2576 mice, the brains of mice infected with ME49 strain were harvested at 3 months post-infection and minced to isolate cysts. Cysts were then isolated under a microscope and 15 were inoculated intraperitoneally into each experimental mouse. Infected mice were raised under SPF conditions at Seoul National University College of Medicine. All behavioral experiments were conducted at 6 months post-infection in 9-month mice, which were then sacrificed for histological examinations and cytokine analysis.

### Water-maze Test

The experiment was conducted using a circular water tank (140 cm in diameter, 45 cm high) filled with 21–23°C water opacified with dry milk powder. The tank contained an invisible platform placed 1.5 cm below the surface of the water. Prominent extra-maze cues were provided to aid platform location. Data were automatically collected using a computerized motion analyzer (Ethovision, Noldus Information Technology H.V., Wageningen, The Netherlands). For descriptive data collection, the pool was subdivided into four equal imaginary quadrants; a video analyzer was used to produce images of swimming animals. Preliminary spatial training was conducted using five time trials of 60 s per trial. During each training session, times taken to find the platform were recorded. Forty-eight hours after the final trial, a spatial probe trial was conducted in the same tank with the platform removed. Mice were allowed to swim for 60 s. Times spent and path lengths in the target quadrant were recorded. Data were automatically analyzed using a video image motion analyzer (Ethovision).

### Y-maze Test

Spatial memory was also assessed using the Y-maze test. The apparatus used consisted of a black plastic maze with three arms that intersected at 60° (60 cm long, 15 cm high, and 10 cm wide). Vertical metal poles located at the outer perimeter of the maze provided spatial cues. A mouse was placed at the end of one arm and allowed to move freely through the maze for 8 min without reinforcements, such as, food and water. The total numbers of entries into arms, including returns to same arms, were recorded. Alternation was defined as entry into each of the three arms consecutively. The maximum number of alternations was calculated by subtracting two from the total number of arms entered. Percent alternation was calculated by expressing actual alternations as a percentage of maximum alternations. Y-maze behavior test groups were as follows; uninfected wild type (n = 10), infected wild type (n = 5), uninfected Tg2576 (n = 9), and infected Tg2576 (n = 10) groups.

### Congo red staining

Brain sections (10 µm) were deparaffinized and then hydrated using a descending ethanol series. After washing in distilled water, sections were incubated in a freshly prepared, alcoholic, saturated alkaline sodium chloride reagent (2.5 mM NaOH in 80% alcohol) for 20 min at room temperature. Slides of sections were stained with 0.4% Congo red (W/V) in an alcoholic, saturated alkaline sodium chloride reagent (freshly prepared and filtered just prior to use) for 30 min at room temperature. Sections were washed in distilled water, counterstained with methyl green for 5 min, dehydrated using an ascending ethanol series, and treated three times with 100% reagent-grade ethanol. Sections where then treated with xylene and coverslipped with Permount (Fisher Scientific Co., Pittsburgh, PA, USA).

### Immunostaining of the pathology of Alzheimer's disease

All animals were anesthetized with Zoletil (15 mg/kg) and Rompun (5 mg/kg) and then perfused with a 4% paraformaldehyde solution for 20 min. Brain tissues were dehydrated through an ethanol gradient, embedded in paraffin, and sectioned at 10 µm. Prior to immunostaining, sections on slides were deparaffinized in an oven, immersed in xylene, and rehydrated using an ethanol gradient. Antigen retrieval in paraffin-embedded tissues was achieved by treating sections with citric acid (1.8 mM citric acid and 8.2 mM sodium citrate) for 10 min and cooling to room temperature. Thirty minutes later, sections were washed with 0.05 M Tris-buffered saline (TBS) and incubated with primary 6E10 antibodies in TBS containing 0.5% BSA and 0.5% Triton X-100 overnight at 4C°. They were then washed three times with TBS and incubated with biotinylated secondary antibodies (Vectastain Elite ABC kit; Vector Laboratories, Burlingame, CA, USA) for 45 min at room temperature. Sections were then reacted with avidin-biotin-peroxidase complex (Vectastain Elite ABC kit) for 1 h; reactions were detected using 0.05% 3,3-diaminobenzidine tetrahydrochloride (DAB) as a chromogen and 0.01% H2O2 in PBS. Sections were then dehydrated using an alcohol series, cleared in xylene, and coverslipped using Canadian balsam solution (Polysciences Inc., Warrington, PA, USA). Peroxidase-stained sections were examined under a light microscope (PM-20, Olympus, Tokyo, Japan).

To count amyloid plaques in the hippocampus and cortex, photomicrographs were acquired using a color digital camera DFC280 (Leica Microsystems, Wetzlar, Germany) attached to a microscope (BX-51; Olympus). Numbers of amyloid plaques were counted using Image J software (Version 1.45).

### Hematoxylin and eosin staining

Brain tissues were embedded in paraffin and coronally sectioned at 10 µm through the hippocampus, mounted, and stained with hematoxylin and eosin. They were then dehydrated using a graded alcohol series, cleared in xylene, and coverslipped in Canadian balsam solution. Neuronal degeneration in hippocampus was determined by detecting eosinophilic neurons under a light microscope (Olympus PM-20; Olympus). To count degenerative cells in the hippocampal dentate gyrus, photomicrographs were acquired with a color digital camera DFC280 (Leica) attached to a microscope (BX-51; Olympus). Degenerative cells were counted using Image J software (Version 1.45).

### Western blot analysis

Cultured BV-2 cells or brain tissues were homogenized in a solution containing a protease inhibitor cocktail (Roche, Indianapolis, IN) using a potter-Elvehjem homogenizer (OMNI International, Kennesaw, GA, USA). Homogenates were then centrifuged at 1,000 g for 15 min. Proteins were separated by SDS-PAGE and transferred to PVDF membranes, which were then blocked with 5% skim milk in Tris-buffered saline (TBS) for 2 h. Protein bands were confirmed with 6E10 antibody to β-amyloid peptide and horseradish peroxidase-conjugated secondary antibody (Amersham Pharmacia, Buckinghamshire, UK), and immunoreactive bands were visualized using an ECL enhanced chemiluminescence system (ECL; Amersham Pharmacia, Buckinghamshire, UK). β-Actin and GAPDH were used as loading controls.

### *Toxoplasma gondii* lysate antigen

*T. gondii* lysate antigen (TLA) was prepared as previously described with slight modification [44]. Briefly, tachyzoites of *T. gondii* RH strain were obtained from the peritoneal exudates of infected mice. Exudates were passed twice through a 25-gauge needle and then through 5 µm filter membranes to remove debris and host cells. Parasites were then washed, resuspended in phosphate-buffered saline (pH 7.4), and sonicated on ice. The supernatant (TLA) was filter-sterilized through a 0.22 µm membrane, and the protein concentration in TLA was determined using a Nanodrop (Thermo Scientific, Rockford, IL). TLA was stored at −70°C until required.

### In Vitro activation of BV-2 cells using recombinant cytokines

The murine microglial cell line, BV-2, was cultured in Dulbecco's modified essential medium (DMEM; Applied Scientific, San Francisco, CA) containing 5% heat-inactivated fetal calf serum (Hyclone, Ogden, UT), 4 mM L-glutamine, 0.2 mM penicillin, 0.05 mM streptomycin, and 20 mM HEPES (Sigma, St. Louis, MO) at 37°C in a CO_2_ incubator [45]. Cells were then washed twice with serum-free DMEM, and incubated for 6 or 24 h in 96 well culture plates (SPL Lifescience, Pocheon-si, Gyeonggi-do, Korea) in 100 ng/ml lipopolysaccharide (Sigma), 20 ng/ml IL-10 (PeproTech, Rocky Hill, NJ, USA), 20 ng/ml TGF-β (Cell signaling, Danvers, MA, USA), or 50 µg/ml TLA (*T. gondii* lysate antigen). Cells were then harvested for real-time PCR, and culture supernatants were harvested for nitrite content determinations and cytokine ELISA assays.

### Real-time PCR

Total RNAs from brain tissue samples and BV2 cells were isolated using RNeasy kits (QIAGEN, Hilden, Germany); all samples were reverse transcribed using RT premix (Intron, Sungnam, Korea). Real-time PCR was performed using the iQ5 real-time PCR detection system (Bio-Rad, Hercules, CA) and SYBR green was used to detect amplification products, as described previously [43]. The reaction conditions used were; initial denaturation at 95°C for 10 min, 40 amplification cycles [denaturation at 95°C for 10 s, annealing at 56°C for 30 s, and elongation at 72°C for 30 s], followed by one cycle at 72°C for 5 min. Data analysis was performed using iQ™5 optical system software (Bio-Rad). Primer sequences used for GAPDH, IFN-γ, TGF-β, and IL-10 are shown in Table S1.

### Preparation of primary cultured microglia

Microglial cells were prepared from the primary cultures of mouse brains as previously described [46]. Briefly, mixed glial cell cultures were prepared from postnatal day 1–3 C57BL/6 mice. Meninges were removed from cerebral hemispheres, prepared into single cells by trituration, and incubated in glial cell culture medium (DMEM supplemented with 10 mM HEPES, 10% FBS, 2 mM L-glutamine, and 1× antibiotic-antimycotic) in 75-cm^2^ flasks at 37°C in a 5% CO_2_ incubator. The medium was changed every five days, and microglial cells were harvested from mixed glial cultures on culture day 14. After shaking at 200 rpm for 3 h on an orbital shaker, culture media were collected and centrifuged at 800 g for 10 min. Microglia were plated in glial culture media and 20 min later plates were washed with medium to remove unattached astrocytes. The purity of microglia was routinely monitored and was approximately 98.5% as determined by staining with rabbit anti-Iba-1 antibody (1∶1,000 Wako Pure Chemical Industries, Osaka, Japan) (Figure S2).

### Nitric oxide production by primary cultured microglia and BV-2 cells

Culture supernatants of primary cultured or BV-2 microglial cells were assayed for nitrite content, which reflects NO production, using Griess reagent (0.1% naphthylethylene diamine dihydrochloride and 1% sulfanilamide containing 2.5% phosphoric acid in equal volumes), as described previously [45].

### ELISA

Cytokine levels were determined using cytokine ELISA kits (Immuno-Biological Co., Ltd., Takasaki, Japan). Briefly, 100 µl of culture supernatant was added to 96 well plates individually coated with primary antibody against each cytokine and incubated overnight at 4°C. After washing, 100 µl of labeled antibody solution included in the ELISA kit was added and incubated for 1 hr at 4°C in the dark. After washing, the chromogen provided in the kit was added and the mixture was incubated for 30 min at room temperature in the dark. Stop solution was then added and the intensities of color reactions were measured at 450 nm using a microplate absorbance reader (TECAN, Männedorf, Switzerland).

### Passive avoidance task

The passive avoidance task determines the ability of a mouse to remember a foot shock and to avoid learned stimuli [43]. Briefly, apparatus used consisted of a shuttle box divided into two chambers of equal size, one illuminated and one dark, and both equipped with a grid floor. Training begins by placing a mouse in the light chamber opening the door to the dark chamber 20 seconds later. Because most mice prefer the dark chamber, experimental mice quickly enter the dark chamber. Training was repeated until the rats entered the dark compartment within 20 sec (training trial). After the training period, when a mouse entered the dark chamber, the door closed automatically and the mouse was delivered an electric foot shock (0.3 mA, 100 V, 3 s) after all four limbs had entered the dark chamber (acquisition trial). For actual testing, a mouse was placed in the light compartment 24 h after the acquisition trial and the time taken to enter the dark chamber (latency) was measured for 300 sec (retention trial). The following animal groups underwent passive avoidance testing; uninfected wild type (n = 10), uninfected Tg2576 (n = 10), and infected Tg2576 (n = 12) groups.

### Statistical Analysis

Data are presented as means ± SEMs. One-way ANOVA with Tukey's *post-hoc* test was used to assess differences between experimental groups. Statistical significance was accepted for p values of <0.05.

## Supporting Information

Figure S1 **The passive avoidance test was used to confirm the effect of** ***T. gondii*** **infection on memory and behavior in wild type and Tg2576 mice.** Time to enter the dark chamber was measured for 300 sec (retention trial). The time taken for *T. gondii*-infected Tg2576 mice (TG+ME49) was greater than that taken by uninfected Tg2576 mice (TG+PBS).
(TIF)

Figure S2 **Primary cultured microglia obtained from mouse brain.** The purity of microglia used in this study was approximately 98.5% as determined by staining with rabbit anti-Iba-1 antibody.
(TIF)

Table S1 **Primers used for qPCR.**
(TIF)
