# Supplementary figures and images for: Toxoplasma gondii Infection in the Brain Inhibits Neuronal Degeneration and Learning and Memory Impairments in a Murine Model of Alzheimer's Disease
Source: PLoS One. 2012 Mar 21;7(3):e33312. doi: 10.1371/journal.pone.0033312 (PMC3310043; doi:10.1371/journal.pone.0033312)

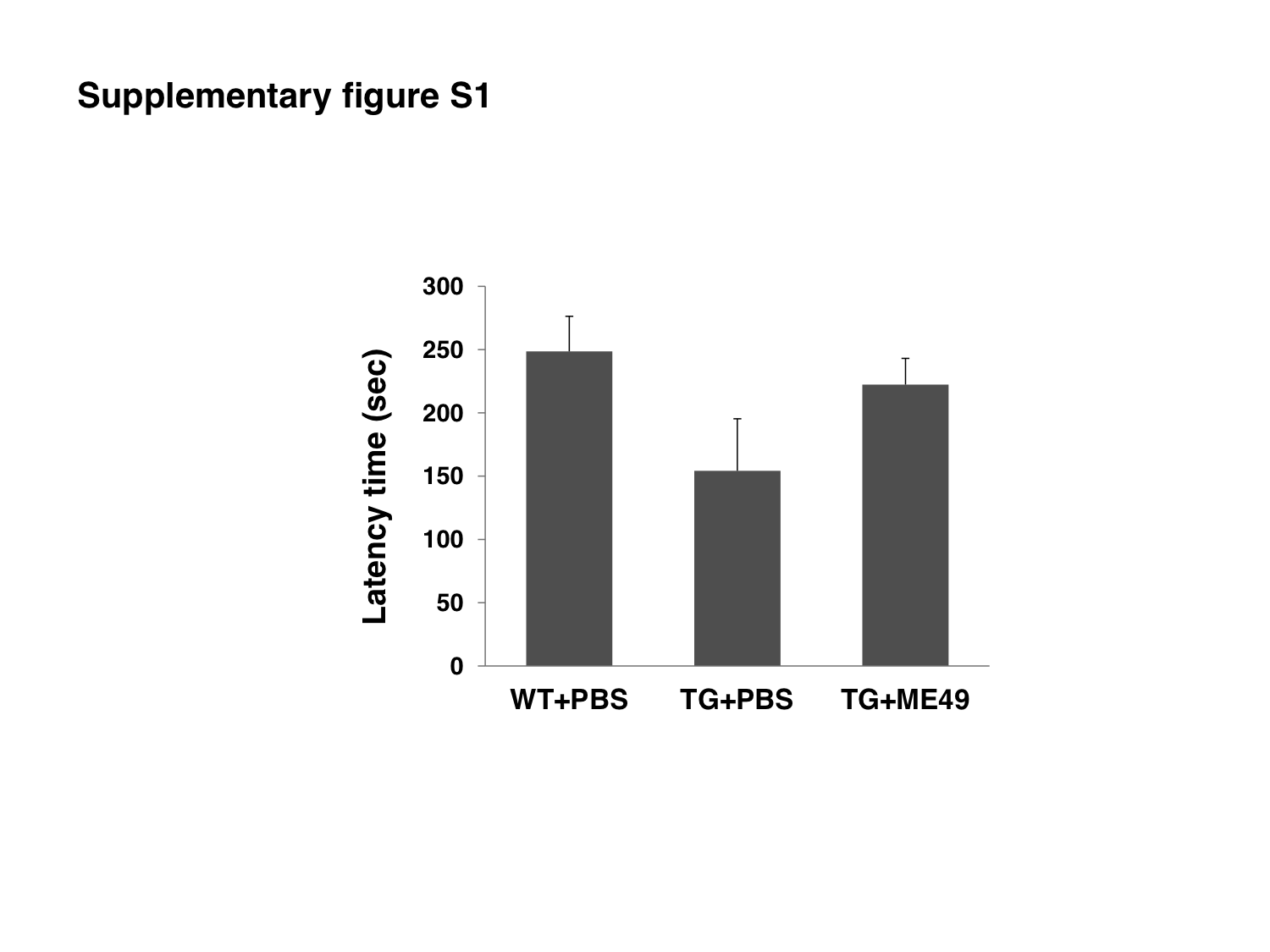

Supplement: Figure S1 — The passive avoidance test was used to confirm the effect of T. gondii infection on memory and behavior in wild type and Tg2576 mice. Time to enter the dark chamber was measured for 300 sec (retention trial). The time taken for T. gondii-infected Tg2576 mice (TG+ME49) was greater than that taken by uninfected Tg2576 mice (TG+PBS). (TIF) [file pone.0033312.s001.tif]

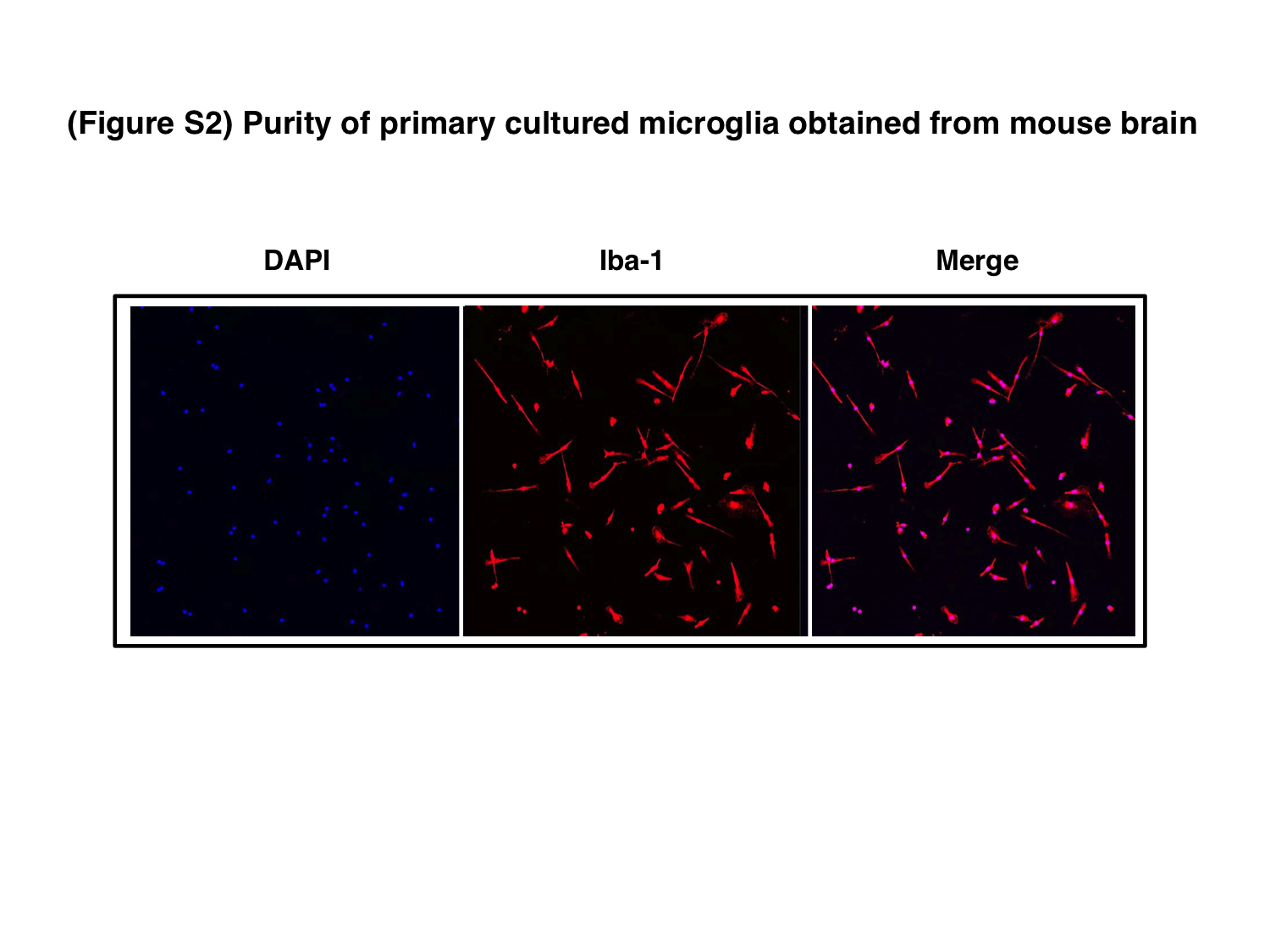

Supplement: Figure S2 — Primary cultured microglia obtained from mouse brain. The purity of microglia used in this study was approximately 98.5% as determined by staining with rabbit anti-Iba-1 antibody. (TIF) [file pone.0033312.s002.tif]

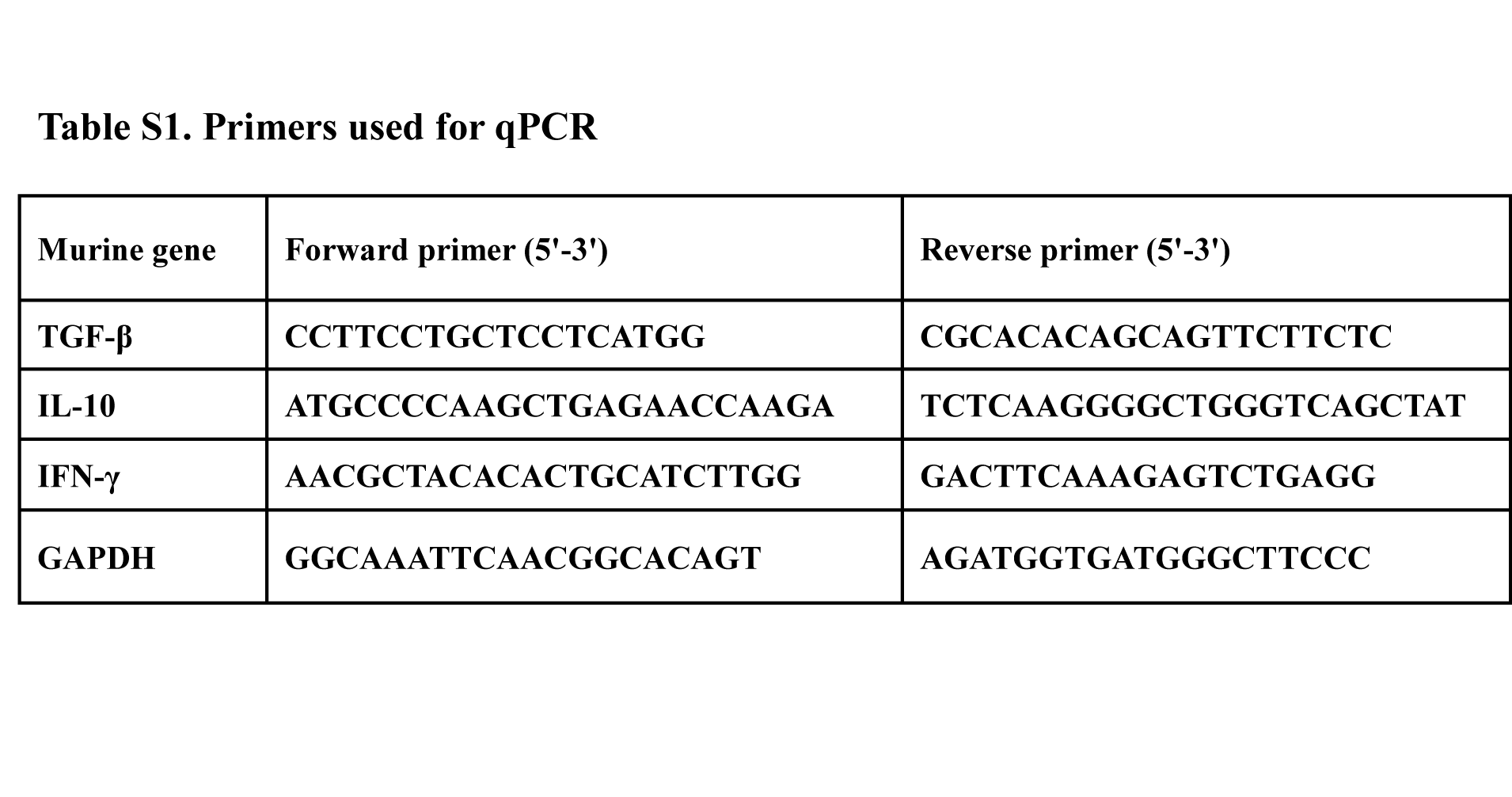

Supplement: Table S1 — Primers used for qPCR. (TIF) [file pone.0033312.s003.tif]
